# 2-(4-Fluoro­phen­yl)-4,5-dimethyl-1-(4-methyl­phen­yl)-1*H*-imidazole

**DOI:** 10.1107/S1600536810022841

**Published:** 2010-06-18

**Authors:** P. Gayathri, J. Jayabharathi, N. Srinivasan, A. Thiruvalluvar, R. J. Butcher

**Affiliations:** a PG Research Department of Physics, Rajah Serfoji Government College (Autonomous), Thanjavur 613 005, Tamilnadu, India; b Department of Chemistry, Annamalai University, Annamalai Nagar 608 002, Tamilnadu, India; c Department of Chemistry, Howard University, 525 College Street NW, Washington, DC 20059, USA

## Abstract

In the title mol­ecule, C_18_H_17_FN_2_, the imidazole ring is essentially planar [maximum deviation of 0.005 (1) Å and makes dihedral angles of 72.33 (8) and 18.71 (8)° with the methyl­phenyl and fluoro­phenyl rings, respectively. The dihedral angle between the two benzene rings is 75.05 (7)°. The crystal packing is stabilized by inter­molecular C—H⋯N hydrogen bonds.

## Related literature

For the optical properties of heterocyclic imidazole derivatives, see: Santos *et al.* (2001 ▶); Huang *et al.* (2004 ▶); Chen & Shi (1998 ▶). For our general experimental procedure for the preparation of imidazoles, see: Jayabharathi *et al.* (2009 ▶).
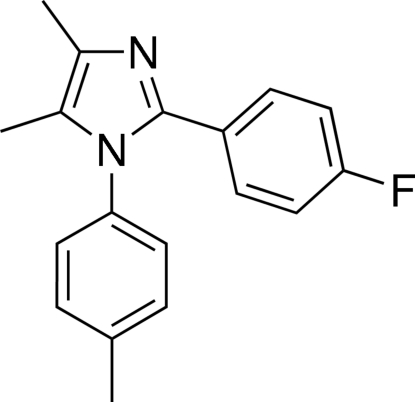


## Experimental

### 

#### Crystal data

C_18_H_17_FN_2_
*M* *_r_* = 280.34
Monoclinic, 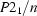

*a* = 9.8888 (2) Å
*b* = 7.6693 (1) Å
*c* = 20.1017 (3) Å
β = 95.915 (1)°
*V* = 1516.40 (4) Å^3^
*Z* = 4
Cu *K*α radiation
μ = 0.65 mm^−1^
*T* = 295 K
0.49 × 0.35 × 0.17 mm

#### Data collection

Oxford Diffraction Xcalibur Ruby Gemini diffractometer
Absorption correction: multi-scan (*CrysAlis PRO*; Oxford Diffraction, 2010 ▶) *T*_min_ = 0.748, *T*_max_ = 1.000
7060 measured reflections
3181 independent reflections
2617 reflections with *I* > 2σ(*I*)
*R*_int_ = 0.018

#### Refinement

*R*[*F* ^2^ > 2σ(*F* ^2^)] = 0.049
*wR*(*F* ^2^) = 0.153
*S* = 1.05
3181 reflections
194 parameters
H-atom parameters constrained
Δρ_max_ = 0.18 e Å^−3^
Δρ_min_ = −0.22 e Å^−3^

### 

Data collection: *CrysAlis PRO* (Oxford Diffraction, 2010 ▶); cell refinement: *CrysAlis PRO*; data reduction: *CrysAlis PRO*; program(s) used to solve structure: *SHELXS97* (Sheldrick, 2008 ▶); program(s) used to refine structure: *SHELXL97* (Sheldrick, 2008 ▶); molecular graphics: *ORTEP-3* (Farrugia, 1997 ▶); software used to prepare material for publication: *PLATON* (Spek, 2009 ▶).

## Supplementary Material

Crystal structure: contains datablocks global, I. DOI: 10.1107/S1600536810022841/si2269sup1.cif

Structure factors: contains datablocks I. DOI: 10.1107/S1600536810022841/si2269Isup2.hkl

Additional supplementary materials:  crystallographic information; 3D view; checkCIF report

## Figures and Tables

**Table 1:** Hydrogen-bond geometry (Å, °)

*D*—H⋯*A*	*D*—H	H⋯*A*	*D*⋯*A*	*D*—H⋯*A*
C12—H12⋯N3^i^	0.93	2.55	3.3714 (19)	148
C16—H16⋯N3^ii^	0.93	2.60	3.5154 (19)	167

Symmetry codes: (i) 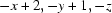; (ii) 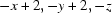.

**Fig. 1.**
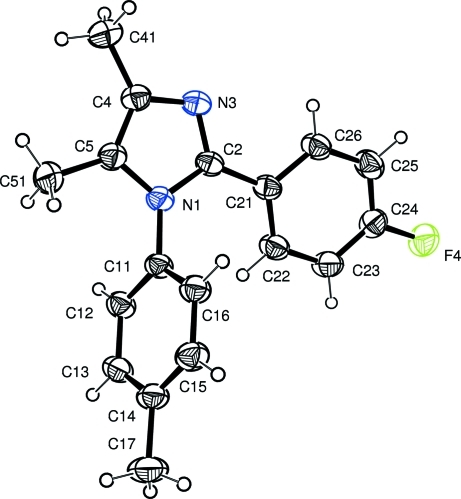
The molecular structure of the title compound, showing the atom-numbering scheme and displacement ellipsoids drawn at the 30% probability level. H atoms are shown as small spheres of arbitrary radius.

**Fig. 2.**
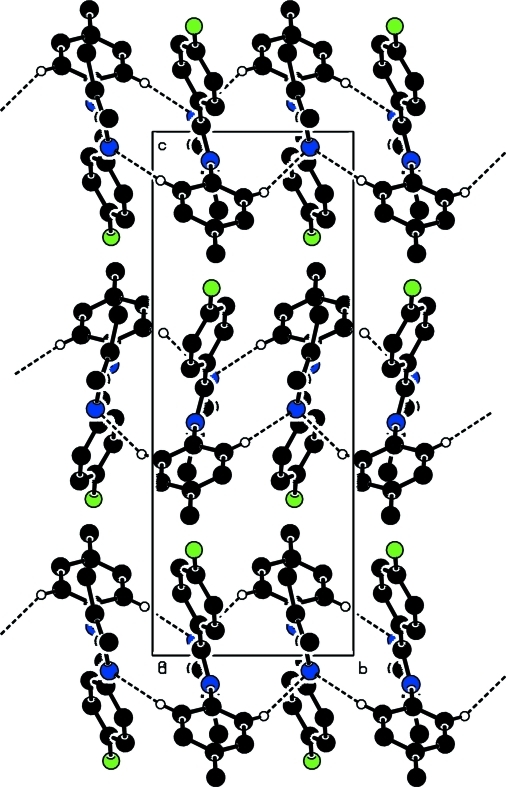
The packing of the title compound, viewed down the a axis. Dashed lines indicate hydrogen bonds. H atoms not involved in hydrogen bonding have been omitted.

## supplementary crystallographic information

### Comment

Imidazole derivatives have been used to construct highly sensitive fluorescent chemisensors for sensing and imaging of metal ions and its chelates in particular those with Ir^3+^ are major components for organic light emitting diodes and are promising candidates for fluorescent chemisensors for metal ions (Santos *et al.*, (2001); Huang *et al.*, (2004) and Chen & Shi (1998)). In this paper we report the crystal and molecular structure of the title compound, a fluorescent chemisensor synthesized in our laboratory.

In the title molecule (Scheme I, Fig. 1), C_18_H_17_FN_2_, the imidazole ring is essentially planar. The imidazole ring makes dihedral angles of 72.33 (8)° and 18.71 (8)° with the methylphenyl (C11–C16) and fluorophenyl (C21–C26) rings respectively. The dihedral angle between the two benzene rings is 75.05 (7)°. The crystal packing is stabilized by C12—H12···N3 (2 - *x*, 1 - *y*, -*z*) and C16—H16···N3 (2 - *x*, 2 - *y*, -*z*) intermolecular hydrogen bonds (Table 1, Fig. 2).

### Experimental

The experimental procedure was used as the same as described in the recent paper (Jayabharathi *et al.*, 2009). The pure biacetyl (1.48 g, 15 mmol) in ethanol (10 ml), *p*-toluidine (1.6 g, 15 mmol), ammonium acetate (7.0 g, 15 mmol) and *p*-fluorobenzaldehyde (1.7 g, 15 mmol) was added about 1 h by maintaining the temperature at 333 K. The reaction mixture was refluxed for 7 days and extracted with dichloromethane. The solid separated was purified by column chromatography using Hexane: Ethyl acetate as the eluent. Yield: 1.93 g (46%).

### Refinement

H atoms were positioned geometrically and allowed to ride on their parent atoms, with C—H = 0.93 – 0.96 Å; *U*_iso_(H) = k*U*_eq_(C), where k = 1.5 for methyl and 1.2 for all other H atoms.

### Crystal data

**Table:**

C_18_H_17_FN_2_	*F*(000) = 592
*M**_r_* = 280.34	*D*_x_ = 1.228 Mg m^−^^3^
Monoclinic, *P*2_1_/*n*	Melting point: 391 K
Hall symbol: -P 2yn	Cu *K*α radiation, λ = 1.54184 Å
*a* = 9.8888 (2) Å	Cell parameters from 4174 reflections
*b* = 7.6693 (1) Å	θ = 4.5–77.3°
*c* = 20.1017 (3) Å	µ = 0.65 mm^−^^1^
β = 95.915 (1)°	*T* = 295 K
*V* = 1516.40 (4) Å^3^	Irregular-plate, colourless
*Z* = 4	0.49 × 0.35 × 0.17 mm

### Data collection

**Table:**

Oxford Diffraction Xcalibur Ruby Gemini diffractometer	3181 independent reflections
Radiation source: Enhance (Cu) X-ray Source	2617 reflections with *I* > 2σ(*I*)
graphite	*R*_int_ = 0.018
Detector resolution: 10.5081 pixels mm^-1^	θ_max_ = 77.6°, θ_min_ = 6.2°
ω scans	*h* = −12→12
Absorption correction: multi-scan (*CrysAlis PRO*; Oxford Diffraction, 2010)	*k* = −8→9
*T*_min_ = 0.748, *T*_max_ = 1.000	*l* = −25→15
7060 measured reflections	

### Refinement

**Table:**

Refinement on *F*^2^	Secondary atom site location: difference Fourier map
Least-squares matrix: full	Hydrogen site location: inferred from neighbouring sites
*R*[*F*^2^ > 2σ(*F*^2^)] = 0.049	H-atom parameters constrained
*wR*(*F*^2^) = 0.153	*w* = 1/[σ^2^(*F*_o_^2^) + (0.0967*P*)^2^ + 0.1202*P*] where *P* = (*F*_o_^2^ + 2*F*_c_^2^)/3
*S* = 1.05	(Δ/σ)_max_ = 0.001
3181 reflections	Δρ_max_ = 0.18 e Å^−^^3^
194 parameters	Δρ_min_ = −0.22 e Å^−^^3^
0 restraints	Extinction correction: *SHELXL97* (Sheldrick, 2008), Fc^*^=kFc[1+0.001xFc^2^λ^3^/sin(2θ)]^-1/4^
Primary atom site location: structure-invariant direct methods	Extinction coefficient: 0.0110 (14)

### Special details

**Table:**

Geometry. Bond distances, angles *etc*. have been calculated using the rounded fractional coordinates. All su's are estimated from the variances of the (full) variance-covariance matrix. The cell e.s.d.'s are taken into account in the estimation of distances, angles and torsion angles
Refinement. Refinement of *F*^2^ against ALL reflections. The weighted *R*-factor *wR* and goodness of fit *S* are based on *F*^2^, conventional *R*-factors *R* are based on *F*, with *F* set to zero for negative *F*^2^. The threshold expression of *F*^2^ > 2σ(*F*^2^) is used only for calculating *R*-factors(gt) *etc*. and is not relevant to the choice of reflections for refinement. *R*-factors based on *F*^2^ are statistically about twice as large as those based on *F*, and *R*- factors based on ALL data will be even larger.

### Fractional atomic coordinates and isotropic or equivalent isotropic displacement parameters (Å^2^)

**Table:**

	*x*	*y*	*z*	*U*_iso_*/*U*_eq_	
F4	0.50712 (11)	0.79381 (18)	−0.20135 (5)	0.0878 (4)	
N1	0.96763 (12)	0.71295 (15)	0.05522 (5)	0.0523 (3)	
N3	1.08318 (12)	0.78452 (16)	−0.02936 (6)	0.0552 (4)	
C2	0.96056 (14)	0.75790 (17)	−0.01134 (6)	0.0506 (4)	
C4	1.17256 (15)	0.7577 (2)	0.02668 (7)	0.0579 (4)	
C5	1.10396 (15)	0.7121 (2)	0.07926 (7)	0.0570 (4)	
C11	0.85905 (13)	0.70419 (18)	0.09791 (6)	0.0508 (4)	
C12	0.82752 (15)	0.5472 (2)	0.12584 (7)	0.0578 (4)	
C13	0.72685 (15)	0.5424 (2)	0.16936 (7)	0.0615 (5)	
C14	0.65756 (14)	0.6921 (2)	0.18449 (7)	0.0620 (5)	
C15	0.69119 (16)	0.8470 (2)	0.15546 (8)	0.0662 (5)	
C16	0.79222 (15)	0.8549 (2)	0.11215 (7)	0.0590 (4)	
C17	0.5499 (2)	0.6850 (3)	0.23290 (10)	0.0871 (7)	
C21	0.83724 (14)	0.76750 (18)	−0.05822 (6)	0.0520 (4)	
C22	0.71458 (16)	0.6883 (2)	−0.04783 (7)	0.0635 (5)	
C23	0.60336 (17)	0.6964 (3)	−0.09575 (8)	0.0695 (5)	
C24	0.61596 (17)	0.7847 (2)	−0.15407 (8)	0.0656 (5)	
C25	0.73553 (18)	0.8635 (2)	−0.16674 (7)	0.0698 (5)	
C26	0.84539 (16)	0.8549 (2)	−0.11881 (7)	0.0617 (5)	
C41	1.32237 (17)	0.7824 (3)	0.02535 (10)	0.0767 (6)	
C51	1.15183 (18)	0.6712 (3)	0.15018 (8)	0.0736 (6)	
H12	0.87286	0.44583	0.11577	0.0693*	
H13	0.70568	0.43696	0.18863	0.0738*	
H15	0.64539	0.94845	0.16502	0.0794*	
H16	0.81419	0.96034	0.09314	0.0708*	
H17A	0.49677	0.79000	0.22908	0.1306*	
H17B	0.49174	0.58636	0.22254	0.1306*	
H17C	0.59289	0.67422	0.27775	0.1306*	
H22	0.70704	0.62892	−0.00804	0.0762*	
H23	0.52178	0.64294	−0.08845	0.0833*	
H25	0.74210	0.92157	−0.20690	0.0837*	
H26	0.92660	0.90820	−0.12686	0.0740*	
H41A	1.36831	0.75508	0.06850	0.1151*	
H41B	1.35437	0.70662	−0.00766	0.1151*	
H41C	1.34053	0.90136	0.01434	0.1151*	
H51A	1.24837	0.68834	0.15761	0.1104*	
H51B	1.10736	0.74684	0.17914	0.1104*	
H51C	1.13064	0.55211	0.15952	0.1104*	

### Atomic displacement parameters (Å^2^)

**Table:**

	*U*^11^	*U*^22^	*U*^33^	*U*^12^	*U*^13^	*U*^23^
F4	0.0716 (6)	0.1229 (10)	0.0657 (6)	0.0028 (6)	−0.0081 (4)	0.0008 (6)
N1	0.0507 (6)	0.0634 (7)	0.0439 (5)	0.0001 (5)	0.0109 (4)	0.0006 (5)
N3	0.0566 (7)	0.0601 (7)	0.0513 (6)	−0.0017 (5)	0.0173 (5)	−0.0018 (5)
C2	0.0560 (7)	0.0543 (7)	0.0435 (6)	−0.0014 (5)	0.0142 (5)	−0.0027 (5)
C4	0.0520 (7)	0.0641 (8)	0.0591 (8)	0.0004 (6)	0.0134 (6)	−0.0024 (6)
C5	0.0529 (7)	0.0664 (8)	0.0525 (7)	0.0029 (6)	0.0089 (6)	−0.0006 (6)
C11	0.0502 (7)	0.0628 (8)	0.0405 (6)	−0.0017 (5)	0.0104 (5)	−0.0018 (5)
C12	0.0636 (8)	0.0619 (8)	0.0494 (7)	0.0013 (6)	0.0138 (6)	0.0003 (6)
C13	0.0631 (8)	0.0723 (9)	0.0507 (7)	−0.0094 (7)	0.0131 (6)	0.0051 (6)
C14	0.0499 (7)	0.0895 (10)	0.0478 (7)	−0.0054 (7)	0.0115 (5)	−0.0045 (7)
C15	0.0618 (8)	0.0740 (9)	0.0652 (8)	0.0081 (7)	0.0184 (7)	−0.0065 (7)
C16	0.0593 (7)	0.0627 (8)	0.0568 (7)	0.0004 (6)	0.0142 (6)	0.0013 (6)
C17	0.0647 (10)	0.1243 (16)	0.0774 (11)	−0.0073 (10)	0.0317 (9)	−0.0054 (11)
C21	0.0568 (7)	0.0584 (7)	0.0423 (6)	−0.0010 (5)	0.0128 (5)	−0.0047 (5)
C22	0.0629 (8)	0.0802 (10)	0.0485 (7)	−0.0113 (7)	0.0113 (6)	0.0024 (6)
C23	0.0582 (8)	0.0922 (11)	0.0589 (8)	−0.0099 (8)	0.0106 (6)	−0.0043 (8)
C24	0.0615 (8)	0.0833 (10)	0.0510 (7)	0.0061 (7)	0.0016 (6)	−0.0076 (7)
C25	0.0779 (10)	0.0850 (11)	0.0463 (7)	−0.0046 (8)	0.0060 (6)	0.0056 (7)
C26	0.0661 (8)	0.0731 (9)	0.0473 (7)	−0.0079 (7)	0.0127 (6)	0.0012 (6)
C41	0.0538 (9)	0.0939 (12)	0.0847 (11)	−0.0009 (8)	0.0182 (8)	0.0034 (9)
C51	0.0647 (9)	0.0993 (12)	0.0561 (8)	0.0042 (9)	0.0029 (7)	0.0074 (8)

### Geometric parameters (Å, °)

**Table:**

F4—C24	1.362 (2)	C23—C24	1.371 (2)
N1—C2	1.3765 (16)	C24—C25	1.375 (2)
N1—C5	1.3845 (19)	C25—C26	1.377 (2)
N1—C11	1.4435 (17)	C12—H12	0.9300
N3—C2	1.3167 (18)	C13—H13	0.9300
N3—C4	1.3738 (19)	C15—H15	0.9300
C2—C21	1.4642 (19)	C16—H16	0.9300
C4—C5	1.359 (2)	C17—H17A	0.9600
C4—C41	1.497 (2)	C17—H17B	0.9600
C5—C51	1.489 (2)	C17—H17C	0.9600
C11—C12	1.378 (2)	C22—H22	0.9300
C11—C16	1.376 (2)	C23—H23	0.9300
C12—C13	1.392 (2)	C25—H25	0.9300
C13—C14	1.387 (2)	C26—H26	0.9300
C14—C15	1.379 (2)	C41—H41A	0.9600
C14—C17	1.516 (2)	C41—H41B	0.9600
C15—C16	1.393 (2)	C41—H41C	0.9600
C21—C22	1.391 (2)	C51—H51A	0.9600
C21—C26	1.3998 (19)	C51—H51B	0.9600
C22—C23	1.387 (2)	C51—H51C	0.9600
			
F4···H13^i^	2.7800	C41···H51A	2.9200
F4···H15^ii^	2.6300	C51···H41A	2.9000
F4···H51B^iii^	2.7100	C51···H25^vi^	3.0000
N3···C12^iv^	3.3714 (19)	H12···N3^iv^	2.5500
N1···H22	2.8300	H13···H17B	2.5600
N3···H26	2.5500	H13···F4^i^	2.7800
N3···H12^iv^	2.5500	H15···H17A	2.3800
N3···H16^v^	2.6000	H15···F4^ii^	2.6300
C4···C26^v^	3.515 (2)	H16···C2	3.0900
C5···C26^v^	3.439 (2)	H16···N3^v^	2.6000
C11···C22	3.1266 (19)	H17A···H15	2.3800
C12···C51	3.333 (2)	H17B···H13	2.5600
C12···N3^iv^	3.3714 (19)	H22···N1	2.8300
C16···C22	3.472 (2)	H22···C11	2.5400
C16···C21	3.5624 (19)	H22···C12	2.9000
C21···C16	3.5624 (19)	H22···C16	3.0200
C22···C16	3.472 (2)	H23···H41B^vii^	2.4800
C22···C11	3.1266 (19)	H25···C51^iii^	3.0000
C26···C4^v^	3.515 (2)	H26···N3	2.5500
C26···C5^v^	3.439 (2)	H26···C5^v^	3.0900
C51···C12	3.333 (2)	H41A···C51	2.9000
C2···H16	3.0900	H41A···H51A	2.3100
C5···H26^v^	3.0900	H41B···H23^viii^	2.4800
C11···H51C	3.0700	H51A···C41	2.9200
C11···H51B	2.8200	H51A···H41A	2.3100
C11···H22	2.5400	H51B···C11	2.8200
C12···H51C	3.0000	H51B···F4^vi^	2.7100
C12···H22	2.9000	H51C···C11	3.0700
C16···H22	3.0200	H51C···C12	3.0000
			
C2—N1—C5	106.89 (11)	C13—C12—H12	120.00
C2—N1—C11	128.55 (11)	C12—C13—H13	119.00
C5—N1—C11	123.33 (10)	C14—C13—H13	119.00
C2—N3—C4	106.51 (12)	C14—C15—H15	119.00
N1—C2—N3	110.50 (12)	C16—C15—H15	119.00
N1—C2—C21	126.42 (12)	C11—C16—H16	120.00
N3—C2—C21	123.00 (11)	C15—C16—H16	121.00
N3—C4—C5	110.22 (13)	C14—C17—H17A	109.00
N3—C4—C41	121.39 (14)	C14—C17—H17B	109.00
C5—C4—C41	128.38 (14)	C14—C17—H17C	109.00
N1—C5—C4	105.87 (12)	H17A—C17—H17B	109.00
N1—C5—C51	122.51 (13)	H17A—C17—H17C	109.00
C4—C5—C51	131.61 (14)	H17B—C17—H17C	109.00
N1—C11—C12	119.76 (12)	C21—C22—H22	119.00
N1—C11—C16	119.15 (12)	C23—C22—H22	119.00
C12—C11—C16	121.04 (13)	C22—C23—H23	121.00
C11—C12—C13	119.07 (14)	C24—C23—H23	121.00
C12—C13—C14	121.17 (14)	C24—C25—H25	121.00
C13—C14—C15	118.31 (13)	C26—C25—H25	121.00
C13—C14—C17	120.45 (15)	C21—C26—H26	119.00
C15—C14—C17	121.24 (15)	C25—C26—H26	119.00
C14—C15—C16	121.45 (14)	C4—C41—H41A	109.00
C11—C16—C15	118.97 (14)	C4—C41—H41B	109.00
C2—C21—C22	124.23 (12)	C4—C41—H41C	109.00
C2—C21—C26	117.73 (13)	H41A—C41—H41B	109.00
C22—C21—C26	117.96 (13)	H41A—C41—H41C	109.00
C21—C22—C23	121.16 (14)	H41B—C41—H41C	109.00
C22—C23—C24	118.74 (16)	C5—C51—H51A	109.00
F4—C24—C23	119.15 (15)	C5—C51—H51B	109.00
F4—C24—C25	118.75 (14)	C5—C51—H51C	109.00
C23—C24—C25	122.10 (15)	H51A—C51—H51B	109.00
C24—C25—C26	118.74 (14)	H51A—C51—H51C	109.00
C21—C26—C25	121.30 (14)	H51B—C51—H51C	109.00
C11—C12—H12	120.00		
			
C5—N1—C2—N3	−0.05 (15)	C41—C4—C5—N1	177.88 (17)
C5—N1—C2—C21	176.96 (13)	C41—C4—C5—C51	−0.7 (3)
C11—N1—C2—N3	167.43 (13)	N1—C11—C12—C13	177.03 (12)
C11—N1—C2—C21	−15.6 (2)	C16—C11—C12—C13	−0.3 (2)
C2—N1—C5—C4	0.51 (16)	N1—C11—C16—C15	−177.49 (13)
C2—N1—C5—C51	179.20 (15)	C12—C11—C16—C15	−0.1 (2)
C11—N1—C5—C4	−167.78 (13)	C11—C12—C13—C14	0.5 (2)
C11—N1—C5—C51	10.9 (2)	C12—C13—C14—C15	−0.2 (2)
C2—N1—C11—C12	117.62 (15)	C12—C13—C14—C17	−178.96 (15)
C2—N1—C11—C16	−64.96 (18)	C13—C14—C15—C16	−0.2 (2)
C5—N1—C11—C12	−76.77 (17)	C17—C14—C15—C16	178.50 (15)
C5—N1—C11—C16	100.65 (16)	C14—C15—C16—C11	0.4 (2)
C4—N3—C2—N1	−0.43 (15)	C2—C21—C22—C23	−177.15 (15)
C4—N3—C2—C21	−177.56 (13)	C26—C21—C22—C23	−0.4 (2)
C2—N3—C4—C5	0.77 (17)	C2—C21—C26—C25	177.29 (13)
C2—N3—C4—C41	−178.00 (15)	C22—C21—C26—C25	0.3 (2)
N1—C2—C21—C22	−18.3 (2)	C21—C22—C23—C24	−0.1 (3)
N1—C2—C21—C26	164.92 (13)	C22—C23—C24—F4	−179.80 (16)
N3—C2—C21—C22	158.32 (14)	C22—C23—C24—C25	0.7 (3)
N3—C2—C21—C26	−18.4 (2)	F4—C24—C25—C26	179.73 (14)
N3—C4—C5—N1	−0.79 (17)	C23—C24—C25—C26	−0.8 (2)
N3—C4—C5—C51	−179.32 (17)	C24—C25—C26—C21	0.3 (2)

Symmetry codes: (i) −*x*+1, −*y*+1, −*z*; (ii) −*x*+1, −*y*+2, −*z*; (iii) *x*−1/2, −*y*+3/2, *z*−1/2; (iv) −*x*+2, −*y*+1, −*z*; (v) −*x*+2, −*y*+2, −*z*; (vi) *x*+1/2, −*y*+3/2, *z*+1/2; (vii) *x*−1, *y*, *z*; (viii) *x*+1, *y*, *z*.

### Hydrogen-bond geometry (Å, °)

**Table:**

*D*—H···*A*	*D*—H	H···*A*	*D*···*A*	*D*—H···*A*
C12—H12···N3^iv^	0.93	2.55	3.3714 (19)	148
C16—H16···N3^v^	0.93	2.60	3.5154 (19)	167

Symmetry codes: (iv) −*x*+2, −*y*+1, −*z*; (v) −*x*+2, −*y*+2, −*z*.
